# Whole genome sequence analysis of the simulated systolic blood pressure in Genetic Analysis Workshop 18 family data: long-term average and collapsing methods

**DOI:** 10.1186/1753-6561-8-S1-S12

**Published:** 2014-06-17

**Authors:** Yun Ju Sung, Jacob Basson, Dabeeru C Rao

**Affiliations:** 1 Division of Biostatistics, Washington University in St. Louis, School of Medicine, St. Louis, MO 63110, USA

## Abstract

Analysis of longitudinal family data is challenging because of 2 sources of correlations: correlations across longitudinal measurements and correlations among related individuals. We investigated whether analysis using long-term average (average of all 3 visits) can enhance gene discovery compared with a single-visit analysis. We analyzed all 200 replicates of simulated systolic blood pressure (SBP) in Genetic Analysis Workshop 18 (GAW18) family data using both single-marker and collapsing methods. We considered 2 collapsing approaches: collapsing all variants and collapsing low-frequency variants. Analysis using long-term average performed slightly better than SBP measured at a single visit. Collapsing all variants performed much better than collapsing low-frequency variants at *MAP4 *and *FLNB*, which included a common variant with a relatively large effect. For several variants in gene *MAP4*, single-marker analysis also provided high power. In contrast, collapsing only low-frequency variants performed much better for *SCAP, DNASE1L3*, and *LOC152217*, where rare variants in these genes had larger effect than common variants. However, for other causal variants, all approaches provided disappointingly poor performance. This poor performance appeared to occur because most of these causal variants explained a very small fraction of phenotypic variance. We also found that collapsing multiple variants did worse than single-marker analysis for several genes when they contained causal single-nucleotide polymorphisms (SNPs) with both positive and negative effects. Because half of causal SNPs were not found in the annotation file based on the 1000 Genomes Project, we found that power was also affected by our use of incomplete annotation information.

## Background

Hypertension has been a difficult phenotype for gene discovery because it is a multifactorial complex phenotype. Although genome-wide association studies (GWAS) have successfully identified several genetic variants for many complex phenotypes, their reliance on common variants has become a barrier to further progress. Three recent GWAS consortia [1-3] identified 29 common variants associated with blood pressure (BP) and hypertension. However, these variants collectively explain less than 2.5% of BP variance, and most of the genetic variants remain yet to be identified (the "missing heritability"). New high-throughput DNA-sequencing technologies now allow us to seek efficient discovery in both previously identified and novel genes of multiple rare variants with supposedly larger effects on BP and hypertension.

Analysis of longitudinal phenotypes in family data has been a challenge because of 2 sources of correlations: correlations across longitudinal measurements and correlations among related individuals within families. Analysis is often limited to a phenotype measured at a single visit (the first visit is most commonly used), ignoring the phenotype measured at other visits. Long-term average (LTA) of BP measurements has shown to be useful in several genetic epidemiology studies [4,5]. Analysis of LTA is much simpler than multivariate longitudinal analysis while using phenotype measured across all visits.

Genetic Analysis Workshop 18 (GAW18) provided whole genome sequencing (WGS) data in a pedigree-based sample and longitudinal phenotype data for BP and hypertension. We investigated whether analysis using the LTA can enhance gene discovery. Because GAW18 sequence data included mostly rare variants, in addition to single-marker analysis, we applied collapsing methods that assess the combined effect of multiple variants in each genomic region. Analyses were performed without knowledge of the underlying simulation model. However, we used the GAW18 answers in presenting the results.

## Methods

### Genotype and phenotype data

We used all 200 replicates of simulated systolic blood pressure (SBP) phenotype on 847 related individuals. GAW18 provided sequence data of 959 related individuals (either directly sequenced or imputed) of Mexican American heritage for more than 8 million variants. It provided 200 replicates of simulated BP and hypertension phenotypes for 849 individuals. Because the data included 2 monozygotic twin pairs, we excluded one from each twin, reducing the sample to 847.

For each individual, we used an LTA of SBP measurements across all three visits. To evaluate the relative performance of using LTA of SBP, we also analyzed SBP measurement at visit 1 and visit 3 separately. For covariates of SBP at visit 1 (and 3), we used age, sex, smoking, body mass index, and medication use at visit 1 (and 3). For LTA, we used the average values of these covariates.

Because of limited time, we restricted our analysis to chromosome 3 based on the results from the first replicate (because chromosome 3 has the largest signal). Chromosome 3 contained 1,200,643 variants that were polymorphic in all 847 individuals. There were 134 causal variants in 22 genes influencing SBP on chromosome 3.

### Annotation files

To apply the collapsing methods, we used the annotation file that was constructed based on the 1000 Genomes Project (http://www.sph.umich.edu/csg/abecasis/MACH/download/1000G-2010-06.html). The annotation file included 1103 genes on chromosome 3. Because this file did not include all variants observed in the GAW18 data, for all 22 causal genes on chromosome 3, we also manually checked the boundaries of these causal genes using PubMed. If a variant was contained in multiple genes, then the variant was used as part of each gene.

### Statistical analysis

For family-based data, we extended the proportion test of Morris and Zeggini [6] to account for family structure. For the combined effect of multiple rare variants in a genomic region, Morris and Zeggini [6] developed the proportion test that models the phenotype, in a linear regression framework, as a function of the proportion of rare variants at which an individual carries a minor allele. For family data, a linear mixed model with a random polygenic component is commonly used to account for phenotypic correlations among related individuals. ProbABEL [7] implements this mixed model approach. Within a gene, we considered 2 collapsing approaches: (a) collapsing all variants and (b) collapsing variants with minor allele frequency (MAF) less than 0.05. We used R to construct a collapsing test statistics in each gene. We used this collapsing test statistics in conjunction with ProbABEL to account for correlation among related individuals. We also used ProbABEL for single-marker analysis.

To evaluate the performance, we computed power (true-positive) and type I error (false-positive) rates at level 0.05. For each gene, power was computed by the proportion of replicates with *p-*values less than 0.05 over 200 replicates. The overall power was computed by averaging these values across all 22 causal genes. The type I error was computed by averaging these values across all null genes. For single-marker analysis, power was computed at each variant. The overall power and type I error were computed by averaging these values across all 134 causal variants and all null variants, respectively.

## Results

### Performance using single-marker approach

Analysis using LTA consistently performed better than analysis using SBP measured at any single visit. SBP measured at visit 1 performed slightly better than analysis using SBP measured at visit 3. However, all three analyses performed poorly in terms of the overall power. The type I error was about 0.06 (0.057 for SBP1 and SBP3 and 0.059 for LTA), roughly keeping the level 0.05. Overall power across all 134 causal variants on chromosome 3 was about 0.12 (0.118, 0.102, and 0.119 for SBP1, SBP3, and LTA, respectively, as shown in Table 1), which was only about twice as high as the type I error rate.

**Table 1:** Summary statistics of single-marker analysis at 134 causal variants on chromosome 3

	MAF	% Variance SBP	Power SBP1	Power SBP3	Power LTA
Minimum	0.0016	0.00000	0.0050	0.0050	0.0000
1st quartile	0.0049	0.00001	0.0250	0.0250	0.0150
Median	0.0082	0.00001	0.0350	0.0400	0.0350
3rd quartile	0.0636	0.00004	0.0925	0.0800	0.1025
Maximum	0.4947	0.02785	1.0000	1.0000	1.0000
Mean	0.0698	0.00064	**0.1169**	**0.1012**	**0.1179**

*LTA*, long-term average; *MAF*, minor allele frequency; *SBP*, systolic blood pressure.

The bold text indicates the overall power (power averaged across all 134 causal variants.

The poor performance appeared to be driven by very small effect sizes of these causal variants. The phenotypic variance explained by most causal variants was very small: the third quartile was 0.0004% as shown in Table 1. The variants at which the empirical power was greater than 0.5 explained a larger fraction of phenotypic variance (shown in Table 2). The only exception was the variant at 47958037, which explained almost no phenotypic variance but power was over 0.87 using all three phenotypes.

**Table 2:** Causal variants with empirical power over 0

Position	Gene	MAF	% Variance explained	SBP1	SBP3	LTA
47956424	*MAP4*	**0.3777**	**0.01426**	0.995	0.885	0.990
47957996	*MAP4*	0.0301	**0.01486**	1.000	1.000	1.000
47958037	*MAP4*	**0.3420**	<1.0E-5	0.980	0.875	0.970
47973345	*MAP4*	0.0082	0.00049	0.955	0.870	0.975
48040283	*MAP4*	0.0318	**0.02785**	1.000	1.000	1.000
48040284	*MAP4*	0.0131	**0.01105**	0.775	0.515	0.775
48054461	*MAP4*	**0.1187**	0.00030	0.640	0.420	0.670
58109162	*FLNB*	**0.4947**	0.00273	0.580	0.510	0.695
141160882	*ZBTB38*	**0.2108**	0.00022	0.525	0.315	0.550
141162128	*ZBTB38*	**0.2109**	0.00061	0.505	0.310	0.550

*LTA*, long-term average; *MAF*, minor allele frequency; *SBP*, systolic blood pressure.

Bold text for MAF indicates MAF greater than 0.05; Also bold text for proportion (%) of variance indicates that with greater than 0.01.

### Performance using collapsing methods

Collapsing all variants performed better than collapsing low-frequency variants (with MAF <0.05) across all 22 causal genes. Similar to single-marker analysis, collapsing methods using the LTA performed slightly better than analysis using SBP measured at any single visit. Type I errors were 0.07 and 0.06 when collapsing all variants and low-frequency variants, respectively, also roughly maintaining the nominal level 0.05. However, all analyses using collapsing methods performed poorly, similar to analysis using single-marker approach. The highest overall power (0.139) was achieved when collapsing all variants using LTA.

Power at each causal gene varied greatly and depended on whether low-frequency variants were collapsed or not. Within each collapsing approach, results were consistent across three phenotypes (SBP1, SBP3, and LTA), as shown in Table 3. In contrast, when using rare single-nucleotide polymorphisms (SNPs), results were considerably different between 2 collapsing approaches, as shown by low correlations in the upper right block in Table 3.

**Table 3:** Spearman correlation across empirical powers at all causal genes

Correlation		Collapse all			Collapse rare		
		**SBP1**	**SBP3**	**LTA**	**SBP1**	**SBP3**	**LTA**

Collapse all	SBP1		**0.80**	**0.94**	−0.11	0.18	0.04
	SBP3			**0.86**	−0.36	0.01	−0.11
	LTA				−0.14	0.13	0.04

Collapse rare	SBP1					0.78	**0.85**
	SBP3						**0.91**
	LTA						

Bold text indicates correlation greater than 0.8 between empirical powers of two approaches.

*LTA*, long-term average; *SBP*, systolic blood pressure.

To understand how power at causal genes depended on collapsing, we focused on the analysis for LTA phenotype and carefully examined results from 2 collapsing approaches. Because these collapsing methods are developed for identifying rare variants, we also compared the results from single-marker analysis by choosing the best power across all rare SNPs within each gene. Table 4 presents these powers at all 22 causal genes on chromosome 3. We classified these genes into 4 groups. The first group contains 6 genes, including *MAP4 *and *FLNB*, in which collapsing all variants performed much better than collapsing low-frequency variants. When collapsing all variants, the highest power was achieved for gene *MAP4 *(0.99). This is consistent with the results from single-marker approach, in which several variants in *MAP4 *had high power, as shown in Table 2. The second highest power was achieved for gene *FLNB *(0.67), again consistent with single-marker analysis. Except for *TUSC2*, all genes in group 1 included a common variant with a relatively large effect.

**Table 4:** Empirical power at 0.05 using collapsing and single-marker approaches at all 22 causal genes for long-term average phenotype

	Causal genes	Total SNPs		Causal SNPs^1^		% Variance explained^2^		Empirical Power		
		
		Common	Rare^3^	Common	Rare	Common	Rare	Collapse all SNPs	Collapse rare SNPs	Best rare^4^
1	*MAP4*	149	739	3	12	**0.01456**	**0.06336**	**0.99**	0.17	1.00
	*FLNB*	321	628	1	5	**0.00273**	0.00007	**0.69**	0.03	0.20
	*ARHGEF3*	899	1303	2	8	0.00001	0.00006	**0.28**	0.07	0.11
	*TUSC2*	1	9	0	0			**0.21**	0.04	
	*ABTB1*	7	41	2	0	**0.00132**		**0.18**	0.02	
	*NMNAT3*	157	332	3	6	0.00014	0.00017	**0.15**	0.00	0.09

2	*ZBTB38*	175	408	5	4	0.00087	0.00003	0.09	**0.15**	0.14
	*DNASE1L3*	57	79	4	4	0.00005	**0.00026**	0.01	**0.34**	0.08
	*LOC152217*	3	4	1	0	0.00001		0.04	**0.28**	
	*SCAP*	31	174	0	2		0.00004	0.03	**0.51**	0.05
	*BTD*	72	216	0	8		0.00011	0.03	**0.08**	0.07
	*FBLN2*	230	452	1	3	0.00002	0.00006	0.02	**0.06**	0.04

3	*CXCR6*	5	18	0	1		<1.0E-5	0.12	0.03	**0.28**
	*PDCD6IP*	145	318	2	3	0.00025	0.00003	0.04	0.14	**0.15**
	*SUMF1*	236	502	1	2	0.00008	<1.0E-5	0.03	0.06	**0.16**
	*SENP5*	130	275	0	5		0.00007	0.02	0.03	**0.12**
	*PTPLB*	158	329	1	2	0.00002	0.00004	0.02	0.03	**0.10**
	*PPP2R3A*	198	860	1	11	<1.0E-5	0.00010	0.02	0.01	**0.12**

4	*SEMA3F*	51	83	0	2		0.00001	0.05	0.03	0.05
	*TFDP2*	363	852	0	5		0.00005	0.04	0.03	0.04
	*PAK2*	294	516	0	0			0.04	0.02	
	*B4GALT4*	90	126	1	0	0.00002		0.01	0.00	
Overall power								0.139	0.095	
Type I error								0.071	0.057	

Bold type for % variance indicates 3 largest among common variants and 2 largest among rare variants; bold type for empirical power indicates the highest across three analysis options.

^1^Number of causal single-nucleotide polymorphisms (SNPs) is computed based on the used annotation file (not based on the answers).

^2^% Variance explained is computed by adding the percent variance explained by each SNP based on the used annotation file (not based on the answers).

^3^Rare SNPs are defined as SNPs with minor allele frequencies (MAFs) less than 0.5.

^4^Best rare is the best power across all rare variants in a gene.

The second group in Table 4 contains 6 genes, including *SCAP, DNASE1L3*, and *LOC152217*; collapsing only low-frequency variants performed much better than collapsing all variants. Except for *ZBTB38*, rare variants in these genes had larger effect than common variants. The third group in Table 4 also contains 6 genes, including *CXCR6*, in which single-marker analysis performed better than either collapsing approach. Gene *CXCR6 *contained one causal variant, and single-marker analysis performed better. All other genes in group 3 contained causal SNPs with both positive and negative effects, for which collapsing multiple variants did worse than single-marker analysis. The fourth group contained 4 genes, and all methods performed poorly. Although gene *PAK2 *did contain 4 causal SNPs in the Answers, none was used for collapsing approaches because they were outside the gene in the annotation file based on the 1000 Genomes Project.

## Discussion and conclusions

Analysis of longitudinal phenotypes in family data has been a challenge because of 2 sources of correlations: correlations across longitudinal measurements and correlations among related individuals within families. We applied association analysis using LTA approach to simulated SBP phenotype in 847 related individuals in GAW18 family data. Overall, analysis using the LTA performed slightly better than analysis using SBP measured at any single visit.

Collapsing all variants performed much better than collapsing low-frequency variants at *MAP4 *and *FLNB*, which included a common variant with a relatively large effect. For several variants in gene *MAP4*, single-marker analysis also provided high power. In contrast, collapsing only low-frequency variants performed much better for *SCAP, DNASE1L3*, and *LOC152217*, where rare variants in these genes had larger effect than common variants. However, for other causal variants, all approaches provided disappointingly poor performance. This poor performance appeared to occur because most of these causal variants explained very small fraction of phenotypic variance. We also found that collapsing multiple variants did worse than single-marker analysis for several genes when they contained causal SNPs with both positive and negative effects. Although our results are based on sequence data from chromosome 3, we expect that our findings would extend to the GAW18 WGS data.

We adapted Morris and Zeggini's collapsing (burden) test to account for family relationship and applied it to the GAW18 data set of all 847 related individuals. Many GAW18 investigators that analyzed only unrelated individuals (with sample size 142) have observed worse performance. For example, using the most commonly used nonburden test sequence kernel association test for unrelated individuals, overall power across all causal genes was about 0.05, which was almost identical to the type I error rate [8]. Our approach of using the averaged value across multiple measurements reduces variability across measurements and should enhance gene discovery. We have not observed much enhancement in this paper. More sophisticated approaches that fully use all available measurements such as multivariate longitudinal analysis may provide better performance.

To apply collapsing methods that assess the combined effect of multiple variants in a gene, it is necessary to know which SNPs are contained in the gene. GAW18 performed WGS of 1043 individuals of Mexican American heritage with an average 60x sequencing depth, with a goal of finding novel SNPs. However, this creates a problem for applying these rare variant approaches because available annotation information does not contain these novel variants. In particular, among 1458 causal SNPs in the Answers, only 731 SNPs were contained in the annotation file that was constructed based on the 1000 Genomes Project. We found that our results were affected by our use of incomplete annotation information. Although the results and issues that we presented in this paper were based on GAW18 data, they may be shared with other sequencing studies.

## Competing interests

The authors declare that they have no competing interests.

## Authors' contributions

YJS conceived of the study and drafted the manuscript. JB carried out statistical analysis and summarized the results. DCR participated in the design of the study and helped to draft the manuscript. All authors read and approved the final manuscript.
